# Beyond incretins: targeting neurokinin receptors for obesity treatment

**DOI:** 10.1038/s41392-024-02100-y

**Published:** 2025-01-15

**Authors:** Doreen Thor, Simone Prömel

**Affiliations:** 1 Rudolf Schönheimer Institute of Biochemistry, Medical Faculty, Leipzig University, Leipzig, Germany; 2 Institute of Cell Biology, Department of Biology, Heinrich Heine University Düsseldorf, Düsseldorf, Germany

**Keywords:** Endocrine system and metabolic diseases, Endocrine system and metabolic diseases

In a study published recently in *Nature*, Sass, Ma, and colleagues describe the neurokinin 2 receptor (NK2R), a G protein-coupled receptor (GPCR), as a novel regulator of food intake as well as energy expenditure, and develop and characterize selective agonists that effectively activate NK2R to promote weight loss. Most interestingly, the authors bridge the gap between rodents and primates, raising hopes for novel treatment options.^1^

Although obesity cases are continuously rising, thereby contributing to major health problems, long-term effective treatment options are still limited. However, it is well established that GPCRs and their pathways are involved in the regulation of different processes controlling appetite, food intake, or energy homeostasis. Thus, several receptors expressed in neurons governing food intake have been proposed to be promising targets for body weight reduction. However, currently the only GPCR agonists approved for obesity treatment are targeting incretin receptors (receptors for glucagon-like peptide-1 (GLP1R), glucose-dependent insulinotropic peptide (GIP), glucagon (GCGR)) (Fig. 1).^2^ Although the melanocortin receptor MC4R seems to be a logical therapeutic target as well, the use of agonists is limited due to cardiovascular side effects. Only setmelanotide was approved for the treatment of monogenetic obesity caused by POMC, PCSK1, or LEPR deficiency.^2^

**Fig. 1:**
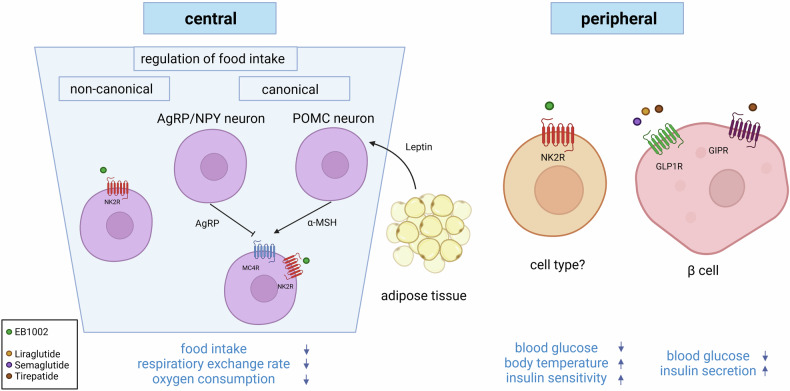
The dual function of NK2R in the regulation of food intake and energy expenditure. Activation of NK2R by the selective agonist EB1002 regulates appetite, food intake, energy expenditure, and metabolic parameters such as insulin sensitivity. The receptor mediates its effects on food intake through neuronal signaling, while its influence on metabolic parameters driving energy expenditure originates mainly from peripheral mechanisms. In the nervous system, NK2R is present on numerous neurons indicating complex underlying mechanisms. However, the regulation of food intake seems to be leptin-independent, but—at least partially—MC4R-dependent. The peripheral regulation of metabolic parameters is different from the ones elicited by incretin receptors as the latter mainly control insulin secretion, although they also have been shown to be active in the brain to, e.g., regulate food intake (not shown for clarity). Created in BioRender. Prömel, S. (2024) https://BioRender.com/z56t410

Due to their central function in regulating metabolic and energy balance pathways and thus, their promising roles as targets in obesity treatment, extensive efforts are undertaken to discover novel GPCRs involved in these processes to unlock new therapeutic possibilities.

Through in-depth analyses of various existing human genetic association data, now NK2R has been identified as a potential regulator of energy homeostasis. Variants of this receptor gene are associated with glycaemic traits and obesity-related characteristics across different human populations. NK2R is part of the neurokinin receptor family, which consists of three members. It is activated by the neuropeptide neurokinin A (NKA) and signals through the Gq pathway.^3^ Found in various tissues, including the ileum and both the central and peripheral nervous systems, it has been linked to intestinal functions, inflammation, and asthma.^4^ As a result, NK2R has been explored as a target for treating conditions such as irritable bowel disease (IBD), colitis, and major depressive disorder. Although three selective NK2R antagonists were identified (saredutant, ibodutant, nepadudant), all failed in phase III clinical trials. With saredutant, developed to treat major depressive disorder, no significant improvement compared to placebo control was observed. Trials for ibodutant and nepadutant to treat irritable bowel syndrome and infant colitis were discontinued for the same reason. Nonetheless, these ventures highlight the pharmacological potential of the receptor. Although NK2R has not yet been associated with the regulation of food intake, the neurokinin receptor NK1R was implicated in this role approximately a decade ago. Specific antagonists for NK1R, which is present in neurons as well as adipocytes, were shown to induce weight loss and improve adipose tissue function in mice. However, although such antagonists are used as pharmaceuticals, none is approved for obesity treatment to date or has been reported for significant weight loss in humans.^5^ Nevertheless, since activation of NK1R might still result in increased weight gain, specific targeting of NK2R is essential.

In diet-induced obese mice, the application of the endogenous NK2R ligand NKA resulted in decreased food intake, body weight, and white adipose tissue amount, while also improving insulin sensitivity. However, as NKA can also activate NK1R^4^ this raises the question of the specificity of the observed effects. A modified neuropeptide (EB1002) selective for NK2R with a longer half-life than NKA produced effects in diet-induced obese mice similar to NKA and showed no effect in mice lacking NK2R. Daily injections of this agonist reduced body weight, adipose depot weights, and food intake, and increased respiratory exchange ratio, oxygen consumption, and fatty acid oxidation. Thereby, weight loss is achieved mainly by decreased fat mass. These findings suggest that NK2R agonism regulates food intake as well as energy expenditure. This specific two-fold control is intriguing and presents a possible dual way of metabolic intervention. The incretin receptor GLP1R for instance, which is the current prime target for obesity treatment, modulates food intake and insulin secretion, but energy expenditure itself remains unaltered.^2^ Subcutaneous versus intracerebroventricular agonist administration reveals that NK2R activation acts via both the periphery and the central nervous system to simultaneously suppress appetite, increase energy expenditure, and improve insulin sensitivity, thereby suggesting two different underpinning mechanisms.

Appetite and food intake regulation by NK2R agonism appears to circumvent leptin signaling but is dependent on MC4R downstream of leptin. In hyperphagic mice lacking the leptin receptor (*ob/ob*), administration of EB1002 reduced food intake in a more pronounced way, while this potentiated effect was not observed in hyperphagic mice lacking MC4R (*Mc4r*). Interestingly, EB1002 was able to overcome refeeding associated with AgRP neuron activation. This could be explained by NK2R present on MC4R neurons overriding the inhibiting signal of AgRP (Fig. 1). However, consistent with previous expression analyses, the agonist also acts through distinct neurons in the dorsal vagal complex (DVC) as well as well as different areas involved in the control of food intake and reward, suggesting complex regulatory circuits. The tissue from which NK2R elicits its role in the regulation of insulin sensitivity and energy expenditure remains to be determined (Fig. 1).

Using this specific agonist, the authors observed no obvious side effects. However, since NK2R antagonists have been previously tested as potential therapeutics ameliorating colitis, bowel disease, or depression, an agonist might exacerbate these conditions. It is worth noting, though, that these antagonists did not prove effective as therapeutics in humans, leaving NK2R agonists as a highly promising avenue.

As NK2R is highly conserved across mammals with a sequence identity of more than 80%, it is conceivable that the effects of the identified NK2R agonists are transferable from mice to higher mammals and ultimately, to humans. Indeed, the administration of the agonists to obese macaques reduced food intake and insulin levels of the animals suggesting similar effects as observed in mice. A decrease in body weight could be monitored, which was more pronounced in diabetic individuals.

Thus, the current study provides a novel and promising opportunity for patients suffering from obesity. Especially, the combined regulation of food intake and energy expenditure and the absence of observable adverse effects provide benefits missing in the incretin therapy. Although it needs to be noted that the weight loss was not as pronounced as observed with the incretin receptor agonists liraglutide or tirzepatide, one highly promising aspect of targeting NK2R is that its weight-reducing effects are also present in diabetic individuals. Thus, combining the positive aspects of NK2R and incretin receptor agonism might further enhance the therapeutic potential.

## References

[1] Sass, F. et al. NK2R control of energy expenditure and feeding to treat metabolic diseases. *Nature* **635**, 987–1000 (2024). 39537932 10.1038/s41586-024-08207-0 PMC11602716

[2] Muller, T. D., Bluher, M., Tschop, M. H. & DiMarchi, R. D. Anti-obesity drug discovery: advances and challenges. *Nat. Rev. Drug Discov.* **21**, 201–223 (2022). 34815532 10.1038/s41573-021-00337-8 PMC8609996

[3] Sun, W. et al. Structural insights into the activation of neurokinin 2 receptor by neurokinin A. *Cell Discov.* **8**, 72 (2022). 35882833 10.1038/s41421-022-00437-8 PMC9325979

[4] Steinhoff, M. S., von Mentzer, B., Geppetti, P., Pothoulakis, C. & Bunnett, N. W. Tachykinins and their receptors: contributions to physiological control and the mechanisms of disease. *Physiol. Rev.* **94**, 265–301 (2014). 24382888 10.1152/physrev.00031.2013 PMC3929113

[5] Karagiannides, I. et al. Substance P as a novel anti-obesity target. *Gastroenterology* **134**, 747–755 (2008). 18325388 10.1053/j.gastro.2007.12.032 PMC2359157

